# Clinical characteristics, management, and outcomes of pulmonary valve myxoma: systematic review of published case reports

**DOI:** 10.1186/s12957-023-02984-0

**Published:** 2023-03-20

**Authors:** Pandit Bagus Tri Saputra, Ayik Rochyatul Jannah, Ihsan Fahmi Rofananda, Makhyan Jibril Al-Farabi, Citrawati Dyah Kencono Wungu, Hendri Susilo, Mochamad Yusuf Alsagaff, Arief Gusnanto, Yudi Her Oktaviono

**Affiliations:** 1 Department of Cardiology and Vascular Medicine, Faculty of Medicine, Universitas Airlangga—Dr. Soetomo General Academic Hospital, Surabaya, Indonesia; 2 Faculty of Medicine, Universitas Airlangga, Surabaya, Indonesia; 3 Department of Physiology and Medical Biochemistry, Faculty of Medicine, Universitas Airlangga, Surabaya, Indonesia; 4 Institute of Tropical Disease, Universitas Airlangga, Surabaya, Indonesia; 5 Department of Cardiology and Vascular Medicine, Universitas Airlangga Hospital, Surabaya, Indonesia; 6 School of Mathematics, University of Leeds, Leeds, UK

**Keywords:** Pulmonary valve myxoma, Cardiac tumor, Cardiovascular disease, Clinical characteristics, Systematic review

## Abstract

**Background:**

Cardiac myxoma is the most common type of primary cardiac tumor, with the majority located in the atrial wall. The tumor is attached to valvular structures in a few cases, of which the pulmonary valve is the least affected. Pulmonary valve myxoma may have different clinical manifestations from the more common cardiac myxomas because of its vital position. A misdiagnosis of these types of cardiac myxoma may be detrimental to the care and well-being of patients. Therefore, this systematic review aims to define the clinical characteristics of pulmonary valve myxoma and how this differs from a more common cardiac myxoma.

**Methods:**

Employed literature was obtained from PubMed, ScienceDirect, Scopus, Springer, and ProQuest without a publication year limit on August 23, 2022. The keyword was “pulmonary valve myxoma.” Inclusion criteria were as follows: (1) case report or series, (2) available individual patient data, and (3) myxoma that is attached to pulmonary valve structures with no evidence of metastasis. Non-English language or nonhuman subject studies were excluded. Johanna Briggs Institute checklists were used for the risk of bias assessment. Data are presented descriptively.

**Results:**

This review included 9 case reports from 2237 articles. All cases show a low risk of bias. Pulmonary valve myxoma is dominated by males (5:4), and the patient’s median age is 57 years with a bimodal distribution in pediatric and geriatric populations. The clinical manifestation of pulmonary valve myxoma is often unspecified or asymptomatic. However, systolic murmur in the pulmonary valve area is heard in 67% of cases. Echocardiography remains the diagnostic modality of choice in the majority of cases. Tumor attached to the pulmonary cusps or annulus and extended to adjacent tissues in all cases. Therefore, valve replacement or adjacent tissue reconstructions are required in 77% of cases. The recurrence and mortality are considerably high, with 33% and 22% cases, respectively.

**Conclusions:**

Pulmonary valve myxoma is more common in males with a bimodal age distribution, and its outcomes seem worse than usual cardiac myxomas. Increasing awareness of its clinical symptoms, early diagnosis, and complete myxoma resection before the presence of congestive heart failure symptoms are important in achieving excellent outcomes. A firm embolization blockade is needed to prevent myxoma recurrence.

**Supplementary Information:**

The online version contains supplementary material available at 10.1186/s12957-023-02984-0.

## Background

Cardiac myxoma is the most common primary heart tumor, comprising 75% of all cardiac tumors [1]. The prevalence of cardiac myxoma ranges from 0.001 to 0.03% in the general population in several autopsy series [1, 2]. Cardiac myxoma is more common in females than males [1]. Cardiac myxoma can affect in some area in the heart such as the left atrium (75%), right atrium (15–20%), left ventricle (3–4%), and right ventricle (3–4%) [3]. The majority of cardiac myxomas are found in left atrial chambers, although it arises from cardiac valves in some cases, which are known as valvular myxoma [4, 5]. Mitral valve myxoma is the most common valvular myxoma, followed by tricuspid, aortic, and pulmonary valve myxoma [4, 5]. Valvular myxoma may have different characteristics from myxoma in the cardiac chamber due to its vital position and valvular involvement [6, 7].

Myxoma presentations can be categorized into cardiac obstructive symptoms (e.g., syncope, palpitation), constitutional symptoms (e.g., weight loss, low-grade fever), and embolic symptoms [8]. Symptoms depend on the size, location, mobility, invasiveness, and friability of the tumor [9]. Theoretically, a tumor in small orifices, such as the pulmonary valve, causes more severe manifestations of cardiac obstructions than tumors in larger chambers, such as the atria or ventricles. Additionally, the small diameter of the pulmonary orifice creates higher blood velocity that potentially leads to tumor dislodgement, causing acute pulmonary emboli and sudden death. Moreover, valvular attachment or infiltration itself may damage pulmonary valvular structures and require different treatment (e.g., valve replacement). Thus, the clinical characteristics of pulmonary valve myxoma, as well as its outcome, are probably different from other types of cardiac myxoma. However, little is known about pulmonary valve myxoma, and information is mostly sourced from case reports. Aggregated case reports or series show similar results to a systematic review of clinical studies in the setting of extremely rare diseases [10]. Aggregated evidence will provide a better understanding of this disease, thereby improving clinical outcomes. This systematic review aims to describe the clinical characteristics, management, and outcomes of pulmonary valve myxoma in published case reports.

## Methods

The present review followed the Preferred Reporting Items for Systematic Reviews and Meta-Analyses (PRISMA) 2020 [11]. No ethical approval was required as no patients directly participated in this study and all the used data have already been published.

### Eligibility

We performed a systematic search on case reports and a case series of pulmonary valve myxomas that described individual patient data. A pulmonary valve myxoma is defined as a cardiac myxoma that is partially or fully attached to any part of the pulmonary valve. Studies reporting indirect valve destruction due to myxoma that is not attached to the pulmonary valve or mobile tumors are excluded. The publication year has no limitation. Any studies written in languages other than English, those with no available full text and with nonhuman subjects were excluded. Duplicate articles were resolved before the title and abstract screening.

### Search strategy and selection of studies

We conducted a comprehensive systematic database search on August 23, 2022, in PubMed, ScienceDirect, Scopus, Springer, and ProQuest. Relevant articles may be included by manual search or bibliography search to increase the coverage of this extremely rare case. The keyword “pulmonary valve myxoma” was used in the search. Titles and abstracts of the articles to identify potentially eligible studies were independently screened for full-text review.

### Article extraction

We independently extracted relevant articles from the included studies using a structured and standardized form. The following information was extracted: first authors’ name, publication year, and country, patients’ age, gender, presenting symptoms and signs, murmur types, comorbidities or significant medical history, imaging modalities used, electrocardiogram (ECG) changes, histopathology examination, primary valve involvement, tumor size, complications, valvular status, management, and follow-up results.

### Quality assessment

We independently assessed the risk of bias in included studies using the Joanna Briggs Institute (JBI) critical appraisal checklist for case reports and cases [12, 13]. The result of the bias assessment using the JBI checklist was presented as a checklist instead of an accumulated score [12].

### Statistical analysis

Meta-analysis could not be performed because this systematic review includes an extremely rare disease based on published cases. However, the extracted data from the cases were put on a sheet and then quantitatively analyzed using descriptive statistics. Similar findings of variables, such as clinical presentation, ECG findings, recurrence, and mortality, are grouped to evaluate their frequency. For example, we tabulated the reported ECG examinations, such as right bundle branch block (RBBB) and right axis deviation (RAD). Then, we stated that RBBB was found in 50% of reported ECG cases if 3 RBBB was found in 6 reported ECG examinations.

## Results

### Study selection

**Fig. 1:**
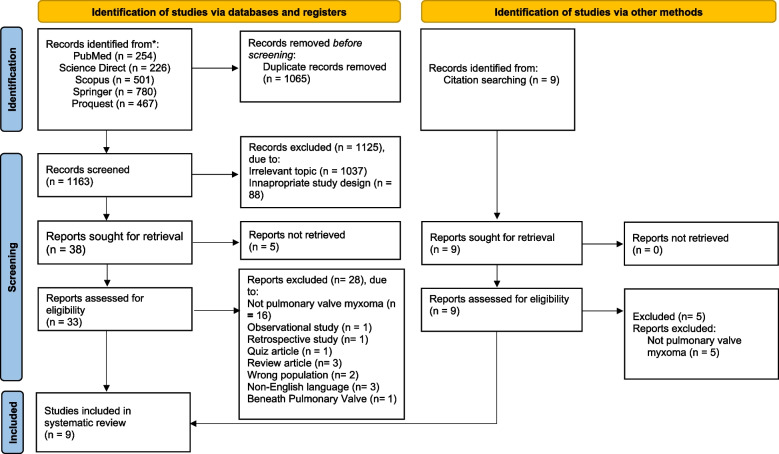
PRISMA flow of study selection

The search result in 2237 records, of which 1065 are duplicates. After title and abstract screening, 1130 articles were excluded. This systematic review included 9 published articles [14–22] after the full-text assessment. PRISMA flow diagram (Fig. 1) presents the process of study selection and the reasons for exclusion. A study is excluded as it is located just beneath the pulmonary annulus (not attached) [23].

### Quality assessment

All included case reports were assessed using a JBI critical appraisal checklist for case reports. The summarized critical appraisal checklist shows that the risks are generally low (Supp. 1) in all case reports, although we do not define the criteria for low-risk bias of the study based on the total score.

### Study characteristics

This systematic review of published cases included 9 case reports [14–22] (Table 1). Out of 9 cases, none of case series of pulmonary valve myxoma was found, indicating the rarity of this disease. The year of published case reports ranged from 1963 to 2022, of which 80% were published in the last 15 years. Most of the published cases are from Asia (6 cases), followed by America (2 cases) and Europe (1 case).

**Table 1:** Demographics, presentation, comorbidities, and diagnosis of pulmonary valve myxoma

No	Author	Country (continent)	Age (year), sex	Presenting symptoms or signs	Murmur	Comorbidities and medical history	Diagnosis imaging	ECG changes	Histopathology
1	Tansel et al. (2011) [20]	Turkey (Asia)	57, F	Progressive dyspnea, palpitation, and chest pain	A grade 3/6 systolic ejection murmur was heard at the left sternal border	None	TTE	Normal	Myxoma
2	Kumagai K et al. (2008) [15]	Japan (Asia)	2, F	Common cold	A grade 3/6 systolic ejection murmur was heard at the left sternal border	None	TTE, CT, and MRI	NA	Myxoma
3	Catton et al. (1963) [22]	USA (America)	0.17, M	Cough, poor intake, vomiting, palpitation, and tachypnea. Congestive heart failure	Grade 3/6 systolic murmur was best heard at the third intercostal space in the left sternal border	Murmur at birth	Angiography	RVH, RBBB, RAE, and wide QRS-T angle	Myxoma
4	Tanabe et al. (2022) [18]	Japan (Asia)	82, F	Asymptomatic in first presentation. Then, congestive heart failure	None	Right femoral neck fracture, hypertension, previous surgery for uterine fibroid	TTE, CT, MRI	Normal	Myxoma
5	Yakirevich et al. (1982) [14]	Israel (Asia)	0.42, M	Intermittent fever, hyperkinesia, anorexia, hepatosplenomegaly, and vomiting	A grade 3/6 systolic murmur was audible over the lower left sternal border and a grade 1/6 diastolic murmur was audible at Erb’s point	Prematurity, low birth weight, and respiratory distress syndrome in first week of life	NA	RVH, RBBB, and RAE	Myxoma
6	Yang et al. (2010) [16]	USA (America)	58, M	Weakness, fevers, chills, and weight loss	Murmur was positive	Heavy alcohol use	TTE	NA	Infected myxoma
7	Sargar et al. (2014) [17]	India (Asia)	66, M	Syncope	None	History of syncope and temporary pacemaker	TTE, CT, MRI	Complete heart block	Myxoma
8	Mai et al. (2012) [21]	China (Asia)	57, F	Paroxysmal frothy cough, chest tightness, intermittent chills, night sweats, weight loss, and fatigue	None	History of paroxysmal cough with a little frothy white sputum	CT	Normal	Myxoma
9	Roux et al. (2017) [19]	Switzerland (Europe)	16, M	Asymptomatic	None	Family history of hypertrophic obstructive cardiomyopathy	TTE	Normal	Myxoma

*CT* CT scan, *F* female, *M* male, *MRI* magnetic resonance imaging, *NA* not available, *TTE* transthoracic echocardiography

### Clinical characteristic

The range of patient age was 2 months to 82 years, with a median of 57 years. The age distribution shows that pulmonary valve myxoma is the most prevalent in pediatric and geriatric populations, with no case observed in ages 20 to 55 years (Table 1). All the cases have been confirmed by histopathology examinations. The male-to-female ratio is 5:4.

Two patients are asymptomatic, in which diagnosis was incidentally established. Additionally, a murmur is revealed in a patient after she is brought to a doctor due to a common cold, which is then diagnosed after an echocardiography examination [15]. Cardiovascular symptoms, such as palpitation, dyspnea, chest pain, syncope, cyanosis, and heart failure, are present in 5 cases (Table 1) [17, 18, 20–22]. Constitutional symptoms are observed in 4 cases [14, 16, 21, 22], of whom 2 had cardiovascular symptoms [21, 22]. Embolic symptoms were not observed in this review. Murmur is present in 5 cases, where it is best heard in the pulmonary valve area during the systolic phase (Table 1) [14–16, 20, 22]. Comorbidities varied between the cases.

Echocardiography, specifically *trans-thoracal* echocardiography, is the most common primary imaging modality used to diagnose myxoma (7 cases), followed by computed tomography (CT) scan (4 cases), magnetic resonance imaging (MRI) (3 cases), and angiography (1 case) (Table 2). Electrocardiography is abnormal in three of seven examinations, commonly presented as right-sided overload, indicated by right ventricle hypertrophy (RVH), RBBB, or right atrial enlargement (RAE) [14, 22]. Additionally, a complete heart block was observed; however, the patient already has it before the myxoma. Evidence and explanation showing complete heart block as the consequence of pulmonary valve myxoma are lacking.

**Table 2:** Tumor location, size, valvular status, complication, surgical procedures, and outcomes

No	Author	Location and extension	Size or diameter (mm)	Complication	Valvular status	Management	Follow-up duration	Recurrence	Deceased or alive
1	Tansel et al. (2011) [20]	Pulmonary valve commissure extending to PA	35 × 26	Moderate pulmonary stenosis and regurgitation	Mass adherent to pulmonary commissure	Myxoma excision and pulmonary arteriotomy	6 months	No	Alive
2	Kumagai K et al. (2008) [15]	Pulmonary annulus and RVOT	24 × 15	Cardiomegaly	NA	Myxoma excision, partial resection of the pulmonary valve, and anterior wall of RVOT Reconstruction of RVOT and PA (transannular patch)	24 months	No	Alive
3	Catton et al. (1963) [22]	Pulmonary valve and RV infundibulum	8 × 10 × 4	Right ventricular hypertrophy, severe pulmonary stenosis, cyanosis, and Eisenmenger syndrome	Abnormal structures in all cusps	Myxoma excision and widening RVOT	7 days	NA	Died
4	Tanabe et al. (2022) [18]	All cusps extended to RV posterior wall and PA	36 × 30	Mild tricuspid regurgitation. Significant PA stenosis	All cusps lumped by the tumor	Myxoma excision and pulmonary valve replacement (bioprosthetic valve)	1 year	Yes	Died
5	Yakirevich et al. (1982) [14]	Pulmonary valves and RV infundibulum	10 × 8	Severe pulmonary valve stenosis	Abnormal structures in all cusps	Myxoma excision and Bougie dilatation of the valve ring	6 months	NA	Alive
6	Yang et al. (2010) [16]	Pulmonary valve and annulus. Extending to RV infundibulum	32 × 40	Right ventricular outflow tract obstruction and endocarditis	NA	Myxoma excision and pulmonary valve replacement (epic tissue valve)	NA	NA	Alive
7	Sargar et al. (2014) [17]	Posterior pulmonary leaflet and RVOT	30 × 30 × 20	Hemorrhagic pericardial effusion due to pacing lead in RV	Abnormal structure in posterior pulmonary valve leaflet	Myxoma and valve excision. Permanent pacemaker implantation	NA	NA	Alive
8	Mai et al. (2012) [21]	Pulmonary valve root and PA	NA	Cyanosis	Abnormal structure in pulmonary valve root	Myxoma excision and reconstruction of RVOT and PA (transannular pericardial patch)	NA	NA	Alive
9	Roux et al. (2012) [19]	Pulmonary valve and RV infundibulum	8 × 10	NA	NA	Myxoma excision	NA	NA	Alive

*NA* not available, *PA* pulmonary artery, *RVOT* right ventricular outflow tract

Tumor diameter ranged from 8 to 40 mm. The tumor is attached to pulmonary cusps (7 cases) [14, 17–22], annulus (1 case) [15], and both (1 case) [16] (Table 2). Tumor extension was observed in all cases: RV (6 cases) [14–17, 19, 22], pulmonary artery (PA) (2 cases) [20, 21], or both (1 case) [18]. All reported studies show an abnormal pulmonary valve structure. Myxoma excision was performed in all cases. Additionally, other procedures were performed, including right ventricular outflow tract (RVOT) reconstruction (3 cases), valve replacement (2 cases), valve ring dilatation (1 case), and PA reconstruction (2 cases). Histopathology reveals an infected pulmonary valve myxoma in one case [16]. The youngest [22] and oldest [18] patients are deceased out of 9 cases. The youngest case is a 2-month-old male infant that has congestive heart failure symptoms, significant stenosis, and mild condition of Eisenmenger syndrome, which could be attributed to the severe RVOT obstruction. His condition has deteriorated following cardiac angiography; therefore, he undergoes surgical intervention that is relatively disadvantageous regarding the tumor size and condition. He passed away 7 h postoperatively [22]. The oldest case is an 82-year-old female with a large-sized myxoma that clumped all three pulmonary cusps and caused significant PA stenosis. Her myxoma surgery is uneventful, but recurrence occurred in her artificial pulmonary valve in 1-year follow-up. Surgery is not performed due to her poor general condition, and she died of right heart failure [18]. Recurrence occurred in one of the three reported cases.

## Discussion

### Epidemiology

Pulmonary valve myxoma is an extremely rare type of cardiac myxoma with only 9 case reports eligible from 5 databases without time limitation, emphasizing this rarity of the disease. Interestingly, pulmonary valve myxoma has a bimodal age-distribution fashion, with pediatric and elderly patients dominating the cases. In contrast, cardiac myxoma is prevalent in geriatric and adult populations, whereas pediatric cardiac myxoma is uncommon [1, 4, 5, 24, 25]. Pediatric cardiac myxoma may arise from residual primitive endocardial tissue [22]. Data from published literature revealed that pulmonary valve myxoma may be dominated by males, whereas cardiac myxoma is dominated by females [1, 4, 5, 24, 25]. However, the gender prevalence in pulmonary valve myxoma remains questionable because of the small sample size.

### Clinical presentation and diagnostic approach

Pulmonary valve myxoma in very young children is characterized by low body weight and systolic murmur at the left sternal border. The murmur may be heard during birth [22]. Both cases show right heart hypertrophy in ECG examination, consisting of RVH, RBBB, and RAE, which are probably caused by severe pulmonary stenosis [14, 22]. Severe pulmonary stenosis may be caused by smaller tumor size and pulmonary orifice ratio in very young children than in adults. Congestive heart failure is present in severe cases and ends up with mortality [22].

Cardiovascular symptoms are the most common specific presentation. This finding is in concordance with previous studies that show cardiac symptoms as the most common specific symptom, present in 47–66% of cases, with heart failure as the two commonest manifestations [4, 5, 8]. Heart failure symptoms are observed in only less than half of the patients with cardiovascular symptoms, and both cases end with death. This may be due to low resistance in the pulmonary circulation; hence, allowing lower pressure is needed to eject flow from the right ventricle to the pulmonary circulation and RV for compensation, such as by RV hypertrophy [14, 22]. Congestive heart failure seems to be a sign of decompensated RV and severe disease in pulmonary valve myxoma. Constitutional symptoms, such as fever, malaise, and weight loss, are probably caused by pro-inflammatory cytokines (e.g., interleukin-1) secreted by myxoma cells [9]. Children with pulmonary valve myxoma all present with poor intake and vomiting; therefore, the myxoma could contribute to growth failure in children. An embolic event was not reported. Embolic symptoms are variably reported in previous studies, ranging from 6 to 41% [4, 5, 8, 25]. Additionally, one-third of patients are asymptomatic (including a patient with a common cold) and diagnosed incidentally. This finding is in concordance with previous studies that show that asymptomatic cases range from 17 to 46%, and the majority are diagnosed by incidental radiology findings [8].

Murmur is a common clinical sign in pulmonary valve myxoma and becomes a precious clue of cardiac-involving disease, especially in patients with no or unspecific symptoms. A murmur is often heard at the pulmonary valve area during the systolic phase in pulmonary valve myxoma. This probably resulted from stenosis due to tumor obstruction during pulmonary orifice opening. Pulmonary stenosis increases RV pressure and causes right-sided heart overload as well as heart failure in the long term. Right ventricle overload causes tricuspid insufficiency, which is observed in two patients [14, 18]. Perforation of RV by a temporary pacemaker may occur due to increased RV pressure or overload [17]. Additionally, an increase in RV may cause tricuspid abnormality, as shown by a murmur in the tricuspid area [14]. Moreover, the most common ECG abnormalities are right-sided heart overload and RBBB [14, 22]. However, ECG seems an insensitive modality and not specific. In general, other laboratory examinations are not sensitive and specific [8].

Transthoracic echocardiography is the most common primary modality to diagnose pulmonary valve myxoma, including asymptomatic cases. Angiography has been the modality of choice before echocardiography is largely introduced in hospitals. Currently, echocardiography is an affordable, noninvasive, and preferred modality to diagnose cardiac myxoma [26, 27]. Echocardiography can delineate cardiac tumor morphology, size, extension, and attachment as well as its hemodynamic manifestation [28]. Transthoracic echocardiography sensitivity can reach up to 90% and even higher in transesophageal echocardiography. However, differentiating between myxoma, thrombus, and vegetation in the pulmonary valves may be difficult. MRI or contrast-enhanced CT scan can be used in doubtful cases to evaluate myxoma complications.

### Management and outcomes

Surgery is the definitive treatment of cardiac myxoma and should be performed without delay after establishing its diagnosis to avoid sudden death. Sudden death can be caused by complete tumor obstruction or dislodgement as well as a potential embolic manifestation in pulmonary valve myxoma. Even small tumor fragment dislodgement can cause significant thrombus and cause a fatal acute pulmonary embolus.

The tumor can be attached to pulmonary cusps or annulus; both conditions cause cusps abnormality in all cases. Tumor excision may involve pulmonary cusps and require valve replacements in several cases because of valvular involvement. All tumors have extended to the surrounding tissue. Excision should involve a large margin of tumor extension as well as its surrounding tissues because the residual tissue may cause recurrence. Thus, additional surgical procedures are needed in almost all cases. Valve replacement has been reported in 22% of cases. In contrast, valve replacement is an additional procedure that is needed only in < 5% of non-valvular myxoma excision [4, 29]. Anticipation of complex surgical procedures may be needed in pulmonary valve myxoma management.

The mortality of pulmonary valve myxoma is considerably high compared to other cardiac myxomas [4, 5, 30]. This may be caused by valvular involvement as well as its narrow orifice location. However, extreme age seems to contribute to high mortality in pulmonary valve myxoma, regarding death cases involving the youngest and oldest patients. Notably, this systematic review did not directly compare the outcomes between pulmonary valve myxoma and other types of myxoma.

Surgery is a definitive treatment of cardiac myxoma, and its outcome is generally excellent with a mortality range of 0.7–3.1% [4, 5, 30]. High mortality in pulmonary valve myxoma in the current review is caused by disadvantageous general conditions to perform surgery, especially CHF, and is complicated by significant pulmonary stenosis. Additionally, a person with extreme age (extremely young or old age) has relatively unstable hemodynamics and higher mortality postoperatively [31]. The location of the pulmonary valve myxoma itself is important, and additional surgical procedure, such as valve excision or adjacent tissue excision, is more often required than in the usual cardiac myxoma. Additionally, recurrence is observed in one case during a year follow-up time, specifically in the second death case, out of three reported data. This percentage is considerably high as the myxoma recurrence rate is commonly low (< 5% in < 5 years of follow-up) [4, 5, 30, 32]. The high recurrence percentage in pulmonary valve myxoma may be caused by the residual tumor tissue, regarding its vital position and extension to RVOT as well as PA, making the excision area become strictly limited and the margin indistinguishable. Cytotransplantation or systemic embolization can also cause myxoma recurrence [18]. Firm embolization blockade (e.g., gauze) can be used to prevent tumor cell embolization to PA.

### Study limitation

The limitation of this systematic review is that it included case reports only with a small number of participants. Cordero et al. [10] reported that aggregating case reports showed similar results to the meta-analysis of a clinical study in the aspect of case reports. A systematic review of published case reports is useful in the setting of rare diseases [33, 34]. The small number of included cases is due to the rarity of this disease, and we anticipated it by not performing statistical analytics that could potentially lead to bias. Additionally, this systematic review did not directly compare the outcomes between pulmonary valve myxoma and other-type myxomas. Instead, the data is presented descriptively, and the reader should be cautious with the conclusion because of the small sample size.

## Conclusions

Pulmonary valve myxoma is an extremely rare disease. It has a bimodal age distribution in pediatric and geriatric populations, with a male majority. Pulmonary stenosis murmur is often a clue for cardiac involvement, especially in asymptomatic or unspecified symptoms. Echocardiography is a noninvasive recommended modality to diagnose pulmonary valve myxoma. Pulmonary valve myxoma considerably has a relatively high number of (1) valves and tissue reconstructions (2), recurrence, and (3) mortality. Early diagnosis and definitive treatment before the presentation of congestive heart failure symptoms may be important in achieving excellent outcomes.

## Supplementary Information

**Additional file 1.** Bias risk assessment of included studies using Joanna Briggs Institut checklist.

## Data Availability

All data are available in the manuscript and separated files as supplementary.

## Abbreviations

cAMP: Cyclic adenosine monophosphate
CT: Computed tomography
IPD: Individual patient data
JBI: Joanna Briggs Institute
MRI: Magnetic resonance imaging
PA: Pulmonary artery
PKA: Protein kinase A
PRISMA: Preferred Reporting Items for Systematic Reviews and Meta-Analyses
RAE: Right atrial enlargement
RBBB: Right bundle branch block
RV: Right ventricle
RVH: Right ventricle hypertrophy
RVOT: Right ventricular outflow tract
